# Ehlers–Danlos Syndrome—Hypermobility Type: A Much Neglected Multisystemic Disorder

**DOI:** 10.5041/RMMJ.10261

**Published:** 2016-10-31

**Authors:** Yael Gazit, Giris Jacob, Rodney Grahame

**Affiliations:** 1 Internal Medicine F and the Institute of Rheumatology, Tel Aviv Sourasky Medical Center, and Sackler Faculty of Medicine, Tel Aviv University, Tel Aviv, Israel; 2 J. Recanati Autonomic Dysfunction Center, Tel Aviv Sourasky Medical Center, Tel Aviv, Israel; 3 Hypermobility Unit, London and Centre for Rheumatology, Division of Medicine, University College London, London, UK

**Keywords:** Disability, Ehlers-Danlos syndrome, hypermobility syndrome, joint hypermobility, multisystemic, neglect

## Abstract

Ehlers–Danlos syndrome (EDS)—hypermobility type (HT) is considered to be the most common subtype of EDS and the least severe one; EDS-HT is considered to be identical to the joint hypermobility syndrome and manifests with musculoskeletal complaints, joint instability, and soft tissue overuse injury. Musculoskeletal complaints manifest with joint pain of non-inflammatory origin and/or spinal pain. Joint instability leads to dislocation or subluxation and involves peripheral joints as well as central joints, including the temporomandibular joints, sacroiliac joints, and hip joints. Soft tissue overuse injury may lead to tendonitis and bursitis without joint inflammation in most cases. Ehlers–Danlos syndrome-HT carries a high potential for disability due to recurrent dislocations and subluxations and chronic pain. Throughout the years, extra-articular manifestations have been described, including cardiovascular, autonomic nervous system, gastrointestinal, hematologic, ocular, gynecologic, neurologic, and psychiatric manifestations, emphasizing the multisystemic nature of EDS-HT. Unfortunately, EDS-HT is under-recognized and inadequately managed, leading to neglect of these patients, which may lead to severe disability that almost certainly could have been avoided. In this review article we will describe the known manifestations of the extra-articular systems.

## INTRODUCTION

The Ehlers–Danlos syndromes (EDSs) constitute a group of inherited disorders of connective tissue characterized by soft hyperextensible skin and joint hypermobility, distinguished by additional connective tissue manifestations.^1^ The Ehlers–Danlos syndrome was first described by Ehlers in Denmark in 1898 and Danlos in Paris in 1908. They published individual case studies with common features of ligamentous laxity and skin hyperextensibility.^2^ Ehlers–Danlos syndrome—hypermobility type (EDS-HT) is considered to be the most common subtype of EDS^3^,^4^ and the least severe one.^3^ It is characterized by joint laxity, soft, stretchy, and often semi-transparent skin, and musculoskeletal complications, without severe complications of arterial dissection or bowel rupture seen in EDS-vascular type,^1^,^5^ and without hemosiderotic scars and molluscoid pseudotumors seen in the EDS-classical type.^1^,^6^ Ehlers–Danlos syndrome-HT, now considered to be indistinguishable if not identical to the joint hypermobility syndrome (JHS), manifests with musculoskeletal complaints, joint instability, and soft tissue overuse injury.^3^,^7^–^12^ Musculoskeletal complaints manifest with joint pain of non-inflammatory origin and/or spinal pain. Joint instability leads to dislocation or subluxation and involves peripheral joints as well as central joints, including the temporomandibular joints (TMJ), sacroiliac joints, and hip joints.^7^–^9^ Soft tissue overuse injury may lead to tendonitis and bursitis^10^,^11^,^12^ without joint inflammation in most cases.^3^,^11^ Although an inflammatory component is rare, EDS-HT carries a high potential for disability^13^ due to recurrent dislocations and subluxations and chronic pain.^8^,^11^,^12^,^14^,^15^ Throughout the years, extra-articular manifestations have been described, including cardiovascular and autonomic nervous system,^16^–^22^ gastrointestinal,^19^,^23^ hematologic,^24^–^26^ ocular,^27^ gynecologic,^19^,^28^–^31^ neurologic,^19^,^25^,^32^,^33^ and psychiatric manifestations,^7^,^8^,^11^,^19^,^34^,^35^ emphasizing the multisystemic nature of EDS-HT. Unfortunately, EDS-HT is under-recognized and inadequately managed,^36^–^38^ leading to neglect of these patients which may lead to severe disability that almost certainly could have been avoided.^39^

## GENERAL CHARACTERISTICS AND MANIFESTATIONS

**Figure 1:**
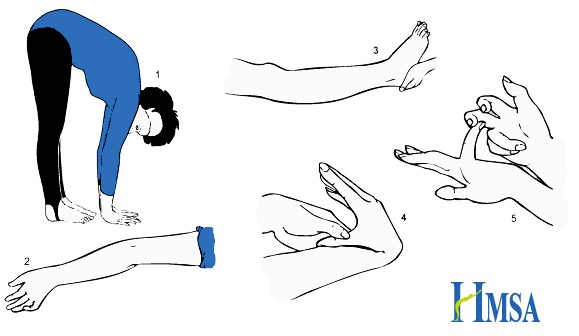
Calculation of the Beighton Score. The Beighton score is calculated as follows:
One point if while standing forward bending you can place palms on the ground with legs straightOne point for each elbow that bends backwardsOne point for each knee that bends backwardsOne point for each thumb that touches the forearm when bent backwardsOne point for each little finger that bends backwards beyond 90 degrees Taken with permission from the Hypermobility Syndromes Association (HMSA) site (http://hypermobility.org/help-advice/hypermobility-syndromes/beighton-score/).

Joint hypermobility (JH), defined as an excessive range of joint movement taking into consideration age, gender, and ethnic background, is inherited^40^,^41^ and may pose no problem. Acquired hypermobility may also result from changes in connective tissue in other diseases such as systemic lupus erythematosus.^42^ Joint hypermobility is recognized by the nine-point Beighton score^43^ (Figure 1) and includes passive dorsiflexion of each fifth finger greater than 90°, passive apposition of each thumb to the flexor surface of the forearm, hyperextension of each elbow greater than 10°, hyperextension of each knee greater than 10°, and ability to place the palms flat on the floor with the knees fully extended.

Ehlers–Danlos syndrome-HT, now considered to be indistinguishable if not identical to the joint hypermobility syndrome (JHS),^44^ is a clinical condition of JH with symptoms of joint instability, arthralgia, myalgia, soft tissue injuries, and arthritis.^45^,^46^ Diagnosis relies on the Brighton criteria (Table 1).^47^,^48^ The predominant presenting complaint is pain, which is often widespread and longstanding, with patients reporting pain ranging from 15 days to 45 years.^39^,^49^ Chronic pain may start in adolescence (with 75% of hypermobile adolescents reporting symptoms by the age of 15) or even as late as the fifth or sixth decade of life.^3^,^39^,^45^ Severity sometimes correlates with the degree of joint instability.^3^,^15^ Fatigue and sleep disturbance, most probably secondary to severe chronic pain, subluxations, and dislocations while changing posture during sleep, are frequently associated.^3^,^11^,^12^,^15^ Affected individuals are often misdiagnosed with chronic fatigue syndrome, fibromyalgia, depression, hypochondriasis, and/or malingering prior to recognition of joint laxity and establishment of the correct underlying diagnosis.^3^ Over the last three decades it has become apparent that EDS-HT has a widespread distribution and is not manifested solely in the joints (Table 2).

**Table 1:** Revised Diagnostic Criteria for Ehlers-Danlos Hypermobility Type, a.k.a. Joint Hypermobility Syndrome (JHS).

Revised Diagnostic Criteria for Ehlers–Danlos Hypermobility Type
Major Criteria: A Beighton score of 4/9 or greater (either currently or historically) Arthralgia for longer than 3 months in four or more joints
Minor Criteria: A Beighton score of 1, 2, or 3/9 (0, 1, 2, or 3 if aged 50+) Arthralgia (>3 months) in one to three joints or back pain (>3 months), spondylosis, spondylolysis/spondylolisthesis Dislocation/subluxation in more than one joint, or in one joint on more than one occasion Soft tissue rheumatism, >3 lesions (e.g. epicondylitis, tenosynovitis, bursitis) Marfanoid habitus (tall, slim, span:height ratio >1.03, upper:lower segment ratio less than 0.89, arachnodactyly (positive Steinberg/wrist signs) Abnormal skin: striae, hyperextensibility, thin skin, papyraceous scarring Eye signs: drooping eyelids or myopia or antimongoloid slant Varicose veins or hernia or uterine/rectal prolapse
JHS is diagnosed in the presence two major criteria, or one major and two minor criteria, or four minor criteria. Two minor criteria will suffice where there is an unequivocally affected first-degree relative.

Taken with permission from the Hypermobility Syndromes Association (HMSA) site (http://hypermobility.org/help-advice/hypermobility-syndromes/the-brighton-score/).^50^

**Table 2:** Multisystemic Nature of EDS-HT.

System	Manifestations
Cardiovascular	Aortic regurgitation, aortic root dilatation, mitral valve prolapse, mitral regurgitation, tricuspid regurgitation, Reynaud phenomenon
Autonomic Nervous System	Palpitations, dizziness, pre-syncope, syncope
Gastrointestinal	Gastroesophageal reflux, dyspepsia, gastritis, delayed gastric emptying, irritable bowel syndrome
Hematologic	Easy bruising, bleeding tendency, prolonged bleeding time, oral mucosal bruises, menometrorrhagia
Ocular	Myopia, strabismus
Gynecologic	Dysmenorrhea, menorrhagia, dyspareunia, uterine prolapse
Urologic	Constipation, fecal soiling, urinary tract infections, urinary incontinence, bladder prolapse, rectal prolapse,
Obstetric	Short labor and delivery, premature rupture of membranes, pelvic pain, varicose veins, worsening of dysautonomia during pregnancy, postpartum hemorrhage, complicated perineal wounds
Neurologic	Headache, local anesthesia failure, postural instability, increased frequency of falls, impaired proprioceptive acuity, Chiari 1 type 1
Psychiatric	Kinesiophobia, anxiety, depression

### Cardiovascular and Autonomic Nervous System Manifestations

A mild degree of aortic root dilatation has been found in up to one-third of EDS-HT patients,^20^,^21^,^22^ necessitating echocardiographic evaluation and surveillance. Raynaud phenomenon was found in 38% of EDS-HT patients.^19^ Patients with EDS-HT may suffer from palpitations, chest pain, dizziness, pre-syncope, and syncope,^17^ which has been attributed in the past to mitral valve prolapse (MVP). Mitral valve prolapse was originally included in the earlier version of the Brighton criteria in 1986.^47^ With more modern evaluation techniques clinically significant MVP has not been found to be more prevalent among EDS-HT patients.^21^,^22^,^50^,^51^ For this reason MVP was removed from the Brighton criteria in 1998.^48^ The frequency of MVP among EDS-HT patients was found to be 28%–67% in more recent studies,^52^,^53^ but its clinical significance is not clear. Symptoms formerly attributed to MVP are now considered to be related to autonomic dysfunction, which was found to be highly prevalent among EDS-HT patients.^16^–^18^

### Gastrointestinal Manifestations

Gastroesophageal reflux was found in 57% of EDS-HT patients.^19^,^23^ Chronic gastrointestinal discomfort was reported in 86% of patients with EDS-HT, attributed to dyspepsia, gastritis, or gastroesophageal reflux. Irritable bowel syndrome was found among 62% of patients. Early satiety and delayed gastric emptying are reported and exacerbated by opioids.^3^

### Hematologic Manifestations

Easy bruising and bleeding tendency is common in all EDS types, including EDS-HT.^25^ It manifests with prolonged bleeding time,^24^,^26^ oral mucosa fragility with mucosal bruises,^9^ and menometrorrhagia.^54^ Since coagulation tests are normal,^24^–^26^ the underlying cause is presumed to be mechanically impaired collagen too weak to afford adequate protection to the capillaries. It is important to note that small and large arterial dissections have not been reported in EDS-HT.

### Ocular Manifestations

Myopia has been found in up to 50% of EDS-HT patients,^54^ and high myopia of more than −6.0 diopters was found in 16% of patients compared with 0% in the control group.^3^,^27^ Strabismus was found in 7% of EDS-HT pediatric patients^55^ (as opposed to only 2%–4% of the general pediatric population), and it is often refractory to surgical correction.^56^ Meyer et al. found size variations and shape abnormalities of collagen fibrils in the extra-ocular muscles that control the movement of the eye.^57^

### Gynecologic Manifestations

Dysmenorrhea and menorrhagia are common^19^,^28^,^29^,^54^,^56^ and thought to be due to muscle contractions occurring with greater force given the loose connective tissue. Dyspareunia was found among 30%–57% of EDS-HT women,^28^,^29^,^58^ thought to be caused by small tears in the vaginal surface and lack of appropriate vaginal secretions.^56^ Pelvic organ prolapse is common,^19^,^28^,^29^,^56^,^59^–^62^ including uterine prolapse which was found in almost 40% of women with EDS-HT.^49^

### Urologic Manifestations

In children with hypermobility constipation and fecal soiling were found to be more common in boys, and urinary tract infection and urinary incontinence more common among girls.^63^ In another pediatric series 13% of girls and 6% of boys suffered from urinary tract infections.^64^ Stress urinary incontinence was found in 40%–70% of women with EDS-HT,^28^,^58^,^65^ often earlier in life, thought to be due to a weakened pelvic floor, which may be worsened to bladder prolapse.^56^ Fecal incontinence was found in up to almost 15% of EDS-HT patients, as compared to only 2.2% of the general population.^65^ Rectal prolapse may also be found among EDS-HT patients.^66^ Furthermore, Dordoni et al. reported on two EDS-HT family members who suffered from visceroptosis, including bilateral kidney prolapse, gastric ptosis, liver prolapse, and ovarian and heart prolapse.^67^

### Obstetric Manifestations

While labor and delivery might be rapid (shorter than 4 hours),^19^,^29^ and premature rupture of membranes is common,^54^,^68^,^69^ pregnancy in women with EDS-HT is generally normal with good maternal and neonatal outcome.^30^,^70^ However, joint laxity and pain may increase during pregnancy.^3^,^29^,^30^,^54^,^70^ Pelvic pain and instability necessitate the use of pelvic belt, crutches, and/or bed rest in 26% of women with EDS, the majority being EDS-HT (compared to only 7% among non-affected women).^56^,^70^ Varicose veins in the legs and the vulva are more common among pregnant women with EDS-HT.^56^

Dysautonomia, characterized by lightheadedness, dizziness, fainting, etc., may worsen during pregnancy,^56^ and when postural orthostatic tachycardia syndrome (POTS) is present a blood pressure fall was reported.^71^ Women with EDS-HT are more prone to postpartum hemorrhage (19% versus 7%) and complicated perineal wounds (8% versus none).^70^ Premature delivery was found to be more related to EDS-HT of the infant (40%), and was less prevalent if the mother had EDS-HT (21%).^70^

### Neurologic Manifestations

A total of 40% of children with EDS-HT^72^ and 50% of adults^14^ suffer from headaches, characterized as chronic recurrent headaches in the absence of structural, congenital, or acquired central nervous system lesions that correlate with their symptoms.^73^ Many complain of headaches related to the neck or facial pain that might be related to jaw or TMJ problems.^56^ Headaches may also be part of dysautonomia, which was found in 78% of EDS-HT patients versus 10% of controls,^17^ characterized by dizziness/ lightheadedness and pre-syncopal episodes, which were found in 88% and 83% of patients, respectively. Partial or complete failure of local anesthesia was described during biopsies and dental or obstetric procedures.^74^,^75^ Hakim and Grahame found local anesthesia resistance in 58% of EDS-HT patients versus 21% of controls.^32^ Proprioceptive acuity has been found to be impaired among EDS-HT adult patients^76^,^77^ and pediatric patients.^78^ Postural instability and balance and gait impairment, resulting in increased frequency of falls, were found among EDS-HT patients as compared to matched healthy controls.^79^ Impaired proprioceptive acuity is thought to influence muscle strength. Therefore, improving muscle strength on the basis of proprioceptive impairment may be more important for reducing activity limitations than just improving muscle strength.^80^ Chiari 1 malformation type 1 was found in 4.7% of EDS-HT patients^19^ and may be associated with cranio-cervical instability and/or the tethered cord syndrome.

### Psychiatric Manifestations

Fear of joint pain and/or instability may lead to avoidance behavior (kinesiophobia) and exacerbate dysfunction and disability.^3^,^7^ Depression and anxiety are more common among EDS-HT patients^7^,^19^,^34^ and are exacerbated by fatigue and pain.^11^,^15^

## GENERAL REMARKS

The multisystemic nature of EDS-HT results in patients having difficulty coping with the syndrome, as well as medical personnel failing to understand the true nature of the condition. This may adversely affect the therapeutic relationship, giving rise to skepticism, resentment, distrust, and hostility on the part of the patient.^3^,^7^

Although EDS-HT is the most common type and the least severe type of EDS, it tends to be underdiagnosed and mistreated, sometimes leading to severe disability that may have been preventable if diagnosed and treated properly.^64^,^81^,^82^ A survey among physiotherapists in the UK found that only 32% of respondents received formal training in EDS-HT management.^83^ Patients perceive a lack of awareness of the syndrome among health professionals and describe delays in diagnosis and access to appropriate health care services.^84^ Many patients reported lengthy diagnosis trajectories and treatment for individual symptoms rather than EDS-HT as a whole. Receiving a correct diagnosis is necessary in order to access appropriate care pathways, for example, referral for physiotherapy for EDS-HT rather than for an acute single joint problem.^84^ A study conducted among military personnel found misdiagnosis of EDS-HT has a disabling impact on military personnel with EDS-HT who are exposed to strenuous physical activities.^85^ Significant neuromuscular and motor development problems have been found among a pediatric population, and delay in diagnosis resulted in poor control of pain and disruption of normal home life, schooling, and physical activities.^64^ Furthermore, they conclude that knowledge of the diagnosis and appropriate interventions are likely to be highly effective in reducing the morbidity and cost to the health and social services.^64^

## DIAGNOSIS

Diagnosis relies on the revised Brighton criteria, but it is important to rule out other connective tissue disorders, especially Marfan syndrome and other types of EDS. Unfortunately, no genetic defect has been found, and for such a prevalent and complex genetic disorder multiple genes might be involved.

## MEDICAL MANAGEMENT

Treatment requires multidisciplinary co-operation and consulting with a cardiologist with echocardiogram monitoring every 2–5 years, orthopedic surgeon with a follow-up once a year, oral and maxillofacial surgeon for temporomandibular joint involvement, gastroenterologist when gastrointestinal manifestations are present, ophthalmologist to rule out other connective tissue diseases and when ocular manifestations are present, urologist and urogynecologist when urologic manifestations are suspected, neurologist and neurosurgeon when prolonged headache is present to rule out Chiari 1, and psychiatry when anxiety and/or depression are suspected. Allergologic consultation may also be needed when there are multiple drug reactions and/or food allergies. An autonomic nervous system specialist should be consulted when signs and symptoms of POTS or other autonomic nervous system manifestations are present. Management includes physiotherapy and hydrotherapy aimed at symmetric and generalized muscle strengthening and proprioception acuity improvement, including deep connective tissue manipulations after each session, occupational therapy when wrists and fingers are involved, and cognitive behavioral therapy for proper adjustment to the chronic nature of the condition. Nutrition has an important role in treating EDS-HT, and nutritional deficiencies should be sought out and treated.

## CONCLUSION

Ehlers–Danlos syndrome-HT is a complex hereditary disorder which is multisystemic, probably due to the prevalence of connective tissue in all body systems. Its gene defect has yet to be found and might be of multigenetic nature, but until then we have to think about the possibility of EDS-HT in every chronic pain patient, and look for joint hypermobility as well as other multisystemic manifestations of this prevalent syndrome.

## Abbreviations

EDS: Ehlers–Danlos syndrome
HT: hypermobility type
JH: joint hypermobility
JHS: joint hypermobility syndrome
MVP: mitral valve prolapse
TMJ: temporomandibular joints
